# Intravitreal bevacizumab role in the treatment of macular edema secondary to retinal vasoproliferative tumor in a patient with neurofibromatosis type 1

**DOI:** 10.3205/oc000045

**Published:** 2016-09-02

**Authors:** Ramin Nourinia, Tahmineh Motevasseli, Zahra Tofighi

**Affiliations:** 1 Ophthalmic research center, Shahid Beheshti University of Medical Science, Tehran, Iran

**Keywords:** macular edema, intravitreal bevacizumab, vasoproliferative mass, neurofibromatosis

## Abstract

**Objective:** To report a case of neurofibromatosis-1 (NF-1) with retinal vasoproliferative tumor (RVPT) and macular edema and exudation that was successfully treated with intravitreal bevacizumab (IVB).

**Method:** A retrospective case report of patient with neurofibromatosis, retinal vasoproliferative tumor and macular edema who received three monthly intravitreal injections of bevacizumab. Optical coherence tomography (OCT) and fluorescein angiography (FAG) before and three months after treatment were done.

**Results:** Macular edema and exudation of the right eye was effectively resolved with IVB injection and vascularity of RVPT significantly decreased after treatment with IVB.

**Conclusion:** Macular edema and exudation secondary to RVPT in patients with NF-1 could be successfully treated with IVB.

## Introduction

Neurofibromatosis type 1 (NF-1) is a multisystem autosomal dominant disorder that is occurred in 1 in 3,000 to 4,000 people worldwide [1]. The clinical manifestations of NF-1 are extremely variable including cutaneous findings, most notably café-au-lait spots and axillary freckling, skeletal dysplasia, and benign and malignant nervous system tumors, most remarkably benign neurofibromas [1]. Common ocular manifestations of NF-1 include optic gliomas and Lisch nodules (iris hamartomas). 

One of the rare ocular complications of NF-1 is a retinal vasoproliferative tumor. A retinal vasoproliferative tumor is an uncommon lesion composed of glial cells and a fine capillary network [2], [3]. For the first time in 1983, Shields et al. reported 12 cases of a retinal vascular mass with distinct clinical features which had not been clearly defined previously [4]. Numerous subsequent publications characterized the clinical and histopathologic features of this tumor, which is currently called retinal vasoproliferative tumor (RVPT). Vision loss may occur secondary to retinal fibrosis, subretinal exudation, and neovascular glaucoma [5]. The pathogenesis and histopathologic characteristics of RVPT have been the subject of controversy. RVPT in patients with NF-1 has previously been reported in only two case reports. In the current study, one NF-1 patient with RVPT who was successfully treated with intravitreal bevacizumab (IVB) is reported.

## Case presentation

A 17-year-old man with NF-1 was referred for evaluation of painless reduction of visual acuity in both eyes since several weeks before. The patient had diffuse cutaneous café-au-lait spots on his abdomen and back. In ocular examination, best corrected visual acuity (BCVA) was 1/10 in the right eye and 1.5 meters counting finger in the left eye, evaluated with standard E-chart at 20 feet distance. Intraocular pressure was 15 mm Hg in both eyes (evaluated with goldman tonometer). Anterior segment examination showed numerous Lisch nodules.

**Figure 1:**
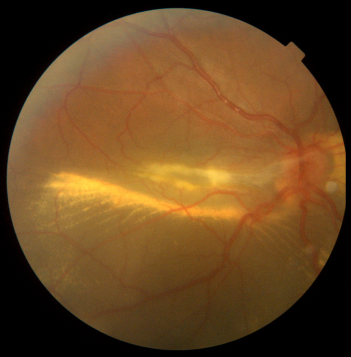
Posterior pole of right eye with subretinal exudation which involved macula with premacular membrane

**Figure 2:**
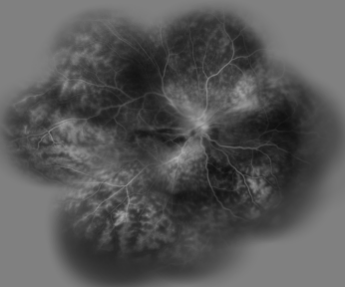
Fluorescein angiogram of the right eye showed diffuse hyper fluorescence which corresponded to telangectatic vessels with leakage

**Figure 3:**
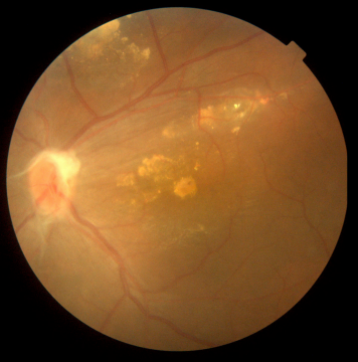
Subretinal old refractile exudation at macula was seen as well as premacular and optic disc fibrous proliferation with macular dragging.

**Figure 4:**
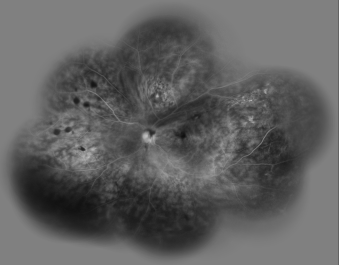
Fluorescein angiogram showed optic disc leakage and diffuse perivascular leakage.

**Figure 5:**
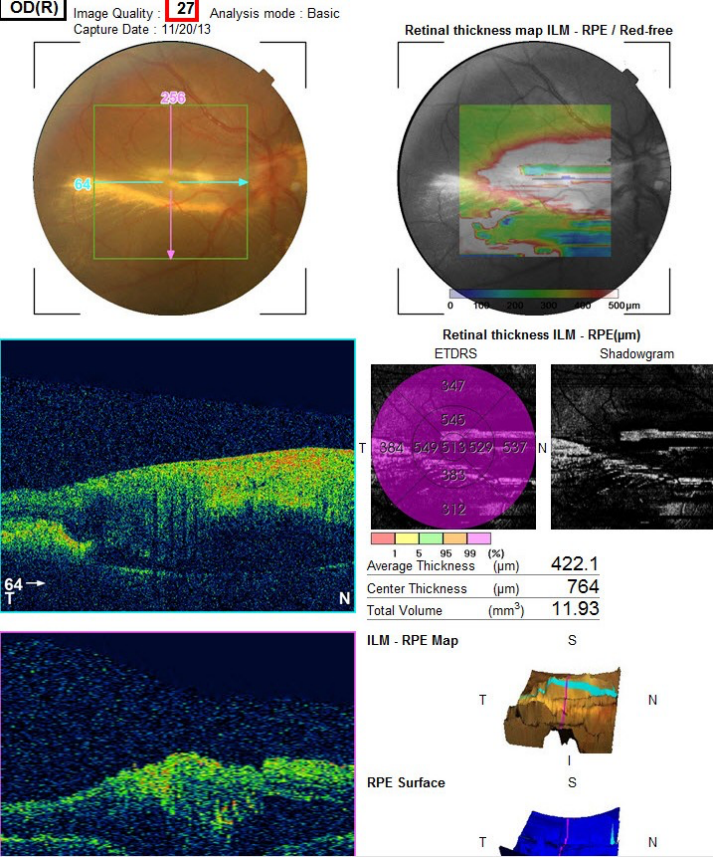
Macular OCT of the right eye with central thickness of 764 µm and subretinal fluid

**Figure 6:**
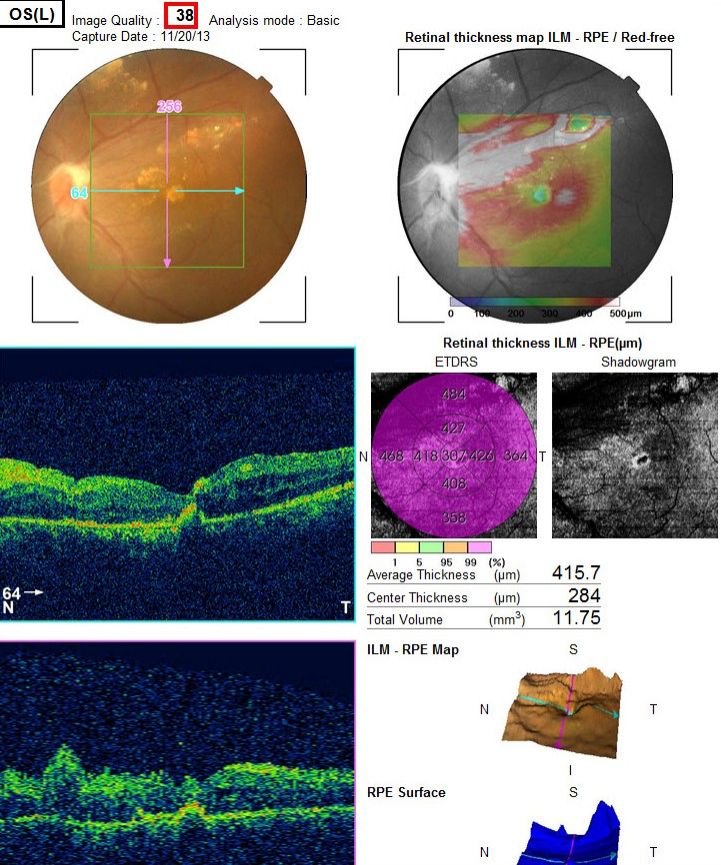
Left eye OCT with macular atrophy and intra and sub retinal hyper-reflective material

**Figure 7:**
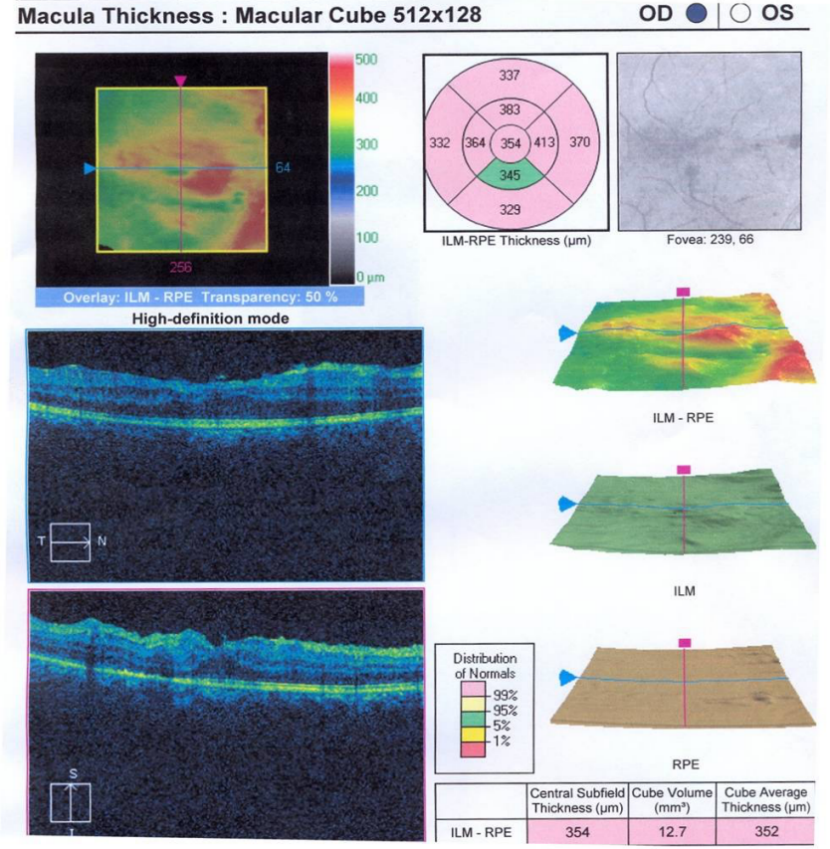
Right eye macular OCT demonstrates significantly reduction of macular thickness after treatment with intravitreal bevacizumab (IVB).

Dilated posterior segment examination of the right eye showed 1+ to 2+ cells in the anterior vitreous and subretinal exudation which involved macula and premacular membrane (Figure 1 (Fig. 1)). Moreover, in the periphery, an elevated pink vascular mass with areas of surrounding subretinal fluid and lipid accumulation was seen. Dilated tortuous feeder vessels, as seen with retinal capillary hemangioma, were not observed. Fluorescein angiogram showed diffuse telangiectatic vessels with leakage (Figure 2 (Fig. 2)). Fundus examination in the left eye showed subretinal old refractile exudations in the periphery and macula as well as premacular and optic disc fibrous proliferation with macular dragging. Fluorescein angiography revealed optic disc leakage and diffuse perivascular leakage (Figure 3 (Fig. 3) and Figure 4 (Fig. 4)). In macular optical coherence tomography (OCT), in the right eye severe macular thickening with central thickness of 764 µm and subretinal fluid were seen, and in the left eye macular atrophy and intra- and subretinal hyper-reflective material were observed (Figure 5 (Fig. 5) and Figure 6 (Fig. 6)). The findings were consistent with vasoproliferative tumor associated with macular edema and retinal exudation. Various therapeutic methods were discussed, and the patient was treated with three monthly injections of bevacizumab (1.25 mg in 0.05mL) in the right eye. After 3 months, macular thickening and subretinal exudation were significantly decreased, retinal detachment overlying the vasoproliferative tumor had resolved, and the tumor appeared less vascularized with fibrotic changes. Macular thickness decreased to 354 µm and vision improved to 3/10 (Figure 7 (Fig. 7)). After 6 months, the condition was stable and the follow-up interval was decided to be 3 months. 

## Discussion

In the present case report, the patient had medium-size RVPT in right eye with massive exudation and macular edema which led to severe visual disturbance. Intravitreal injection of bevacizumab owing to less aggression and less cost for three times was selected as therapeutic choice. After treatment, the size of tumor, macular edema and subretinal exudation were significantly decreased.

NF-1 is a progressive disease, which is manifested as the development of multiple hamartomas originated from neural crest. Retinal tumors, such as astrocytic hamartoma, combined hamartoma of the retina and retinal pigment epithelium, as well as retinal capillary hemangioma are rarely seen in association with NF-1 [6]. Retinal vasoproliferative tumor is also a rare finding in NF-1 which has been reported only in a few cases [2], [3]. This tumor is mostly idiopathic (74%); however, the remained 26% of cases occur in patients suffering from other diseases [5], [7]. RVPT is a red to orange color, nonpigmented lesion. Other presentations of RVPT are comprised of lipoproteinaceus exudates, subretinal fluid, subretinal or even vitreous hemorrhage, vitreous cells, cystoid macular edema, epiretinal membrane, subretinal membrane and hypertrophy of retinal pigmented epithelium [7]. The etiology of PVRT is unknown; however in histopathologic studies, mixed proliferation of glial cells, blood vessels, and retinal pigment epithelial cells has been reported [5]. In addition, other studies suggested that some kind of vasoactive cytokines may play a role in the pathogenesis of RVPT [8]. The response of our patient to intravitreal injection of anti-VEGF agent may support the role of vasogenic factors in the development of RVPT and its complications. 

The management of RVPTs depends on the entire clinical situation and includes simple observation of small asymptomatic lesions. There is no ideal protocol for treatment of RVPTs but various modalities are available with variable success rates [5], [7], including cryotherapy, laser photocoagulation, photodynamic therapy (PDT), brachytherapy, surgical resection, intravitreous anti-VEGF, steroid injections and immunomodulators. All of them can be used alone or in combination [5], [9]. Saito and colleagues showed that vascular endothelial growth factor derived from RVPTs causes retinal neovascularization or exudative retinal changes associated with RVPTs. They believed that intravitreal bevacizumab may be a beneficial therapeutic choice for such patients [10]. The other study described that IVB was advantageous for patients with PVRTs and visual acuity loss of 6/19 and the tumor size of 5.0 mm in diameter and 1.7 mm for its thickness [9]. 

In summary, one of the causes of vision loss in patients with NF-1 could be the subretinal exudation and macular edema secondary to retinal vasoproliferative tumor that may be managed successfully by intravitreal injection of bevacizumab.

## Notes

### Competing interests

The authors declare that they have no competing interests.
